# Preoperative multidisciplinary team meeting improves the incidence of positive margins in pathological T2 prostate cancer

**DOI:** 10.1007/s00345-024-05261-1

**Published:** 2024-10-09

**Authors:** Kohei Kobatake, Keisuke Goto, Yukiko Honda, Miki Naito, Kenshiro Takemoto, Shunsuke Miyamoto, Yohei Sekino, Hiroyuki Kitano, Kenichiro Ikeda, Keisuke Hieda, Akihiro Goriki, Nobuyuki Hinata

**Affiliations:** 1 Department of Urology, Graduate School of Biomedical and Health Sciences, Hiroshima University, 1-2-3 Kasumi, Minami-ku, Hiroshima, 734-8553 Japan; 2 Department of Diagnostic Radiology, Graduate School of Biomedical and Health Sciences, Hiroshima University, 1-2-3 Kasumi, Minami-ku, Hiroshima, 734-8553 Japan

**Keywords:** RARP, MDTM, PSM, Prostatectomy, Multidisciplinary team meeting, Positive surgical margin

## Abstract

**Purpose:**

Positive surgical margins (PSM) after robot-assisted radical prostatectomy (RARP) for prostate cancer (PCa) can increase the risk of biochemical recurrence and PCa-specific mortality. We aimed to evaluate the impact of multidisciplinary team meetings (MDTM) on reducing the incidence of PSM following RARP.

**Methods:**

We retrospectively collected the clinical data of consecutive patients undergoing RARP at Hiroshima University between February 2017 and October 2023. The MDTM, comprising a radiologist, uropathologist, and urologist, reviewed the preoperative magnetic resonance imaging (MRI) and prostate biopsy results of each patient before RARP and considered the areas requiring attention during RARP. Surgeons were categorized as experienced or non-experienced based on the number of RARP procedures performed.

**Results:**

In the pT2 population, the PSM rate was significantly lower in cases evaluated using the MDTM than in those not (11.1% vs. 24.0%; *p* = 0.0067). Cox regression analysis identified that a PSA level > 7 ng/mL (hazard ratio 2.2799) and nerve-sparing procedures (hazard ratio 2.2619) were independent predictors of increased PSM risk while conducting an MDTM (hazard ratio 0.4773) was an independent predictor of reduced PSM risk in the pT2 population. In the pathological T3 population, there was no significant difference in PSM rates between cases evaluated and not evaluated at an MDTM. In cases evaluated at an MDTM, similar PSM rates were observed regardless of surgeon experience (10.4% for non-experienced and 11.9% for experienced surgeons; *p* = 0.9999).

**Conclusions:**

An MDTM can improve the PSM rate of pT2 PCa following RARP.

## Introduction

Positive surgical margins (PSM) are an adverse outcome following robot-assisted radical prostatectomy (RARP) for patients with prostate cancer (PCa) [1]. The concept of PSM, while common in some cancer sites, such as sarcoma, is rare in other common malignancies, such as lung or colorectal carcinoma [2]. In PCa, the presence of a PSM in radical prostatectomy specimens is associated with a greater risk of biochemical recurrence and is an independent predictor of PCa-specific mortality [3, 4]. Thus, patients with PSM require additional treatments, such as adjuvant or salvage radiation therapy, possibly combined with androgen blockade [5, 7]. Conversely, aggressive or advanced diseases may be cured in men with negative surgical margins [6].

The reported overall incidence of PSM ranges from 15 to 29.5% [7, 8, 9, 12], showing significant variability. Especially, PSM in patients with pathological T2 (pT2) and pathological T3 (pT3) are remarkably different (6.8–38.4% in pT2 and 34.0–56.6% in pT3) [9, 10, 14]. Several studies explore the risk factors associated with the development of PSM after RARP such as features related to host, tumor biology, and surgeon [1, 5, 6, 15].

Clinicians should consider PCa sites based on the prostate biopsy [11] and magnetic resonance imaging (MRI) findings. A positive prostate biopsy result at a certain location could predict the PSM status owing to its proximity to the incision site. Moreover, MRI is the gold standard for imaging in PCa [12]. Preoperative MRI features can help estimate the risk of PSMs after radical prostatectomy [13], despite the poor sensitivity of the T stage on preoperative MRI for predicting pathological PSMs.

Our facility accepts referrals for RARP with preoperative MRI from various healthcare organizations; however, MRI scanning conditions and interpretations can vary among facilities. To improve the PSM rate, the authors had the preoperative patient information, including the results of prostate biopsy and prostate MRI, evaluated by a multidisciplinary team meeting (MDTM) before RARP. This study aimed to retrospectively determine the impact of an MDTM on reducing the incidence of PSM following RARP in pT2 and pT3 cases.

## Methods

### Inclusion and exclusion criteria

Patients who received RARP for localized PCs and had undergone preoperative MRI at Hiroshima University Hospital and its affiliated hospitals since February 2017 were included in the study. From July 2022 to October 2023, an MDTM comprising a radiologist with over 15 years of experience in reading MRI, a uropathologist with both 5 years of experience in pathologic diagnosis of urologic cancer and over 15 years of experience as a urologist, and urologists with at least 8 years of experience in urologic surgery at Hiroshima University Hospital has been conducted every week for all cases of planned RARP. The clinical data were retrospectively collected from a consecutive series of patients who underwent evaluation at the MDTM (July 2022–October 2023) and those who did not (February 2017–Jun 2022). The following patients were excluded from the study: patients with unknown preoperative Gleason grade, patients with pT0 disease, and patients who underwent cytoreductive prostatectomy.

### A multidisciplinary team meeting

An MDTM was conducted for patients who were to undergo RARP. The MDTM did not result in a change in the treatment plan in any case. The MDTM participants considered the areas that needed attention during RARP based on a review of the preoperative MRI and prostate biopsy results for each patient. T2-weighted images, diffusion-weighted imaging (DWI), and apparent diffusion coefficient (ADC) maps of the axial and sagittal sections of the prostate MRI were reviewed. Data on the scanning conditions, previous interpretations, and contrast media used for MRI were not collected. Based on the Prostate Imaging Reporting & Data System (PI-RADS) version 2 (14), any findings classified as PI-RADS category 4 or 5 required careful surgical manipulation around the relevant areas, irrespective of whether prostate biopsy results were obtained. In the absence of obvious MRI findings, only the biopsy results were considered for the surgery.

### Surgeons

A total of 19 surgeons performed RARP at random during the observation period. Seven of these surgeons (36.8%) who performed RARP for patients evaluated at an MDTM overlapped, and they also performed RARP for patients without an MDTM. In accordance with the Japanese Urological Association regulations, surgeons who had performed more than 40 RARP procedures and qualified as proctors were defined as experienced surgeons, whereas those who had performed fewer than 40 RARP procedures were defined as non-experienced surgeons.

### Statistics

Univariate and multivariate Cox regression analyses, unpaired t-test, and χ^2^ test were used to compare the two groups. The unpaired t-test and χ^2^ test were performed using GraphPad Prism 8 (GraphPad Software Inc., RRID: SCR_002798). Univariate and multivariate Cox regression analyses were performed using JMP software (SAS Institute Inc., RRID: SCR_008567).

## Results

### PSM in each pT2 and pT3 population

Patient demographics for the pT2 and pT3 populations are respectively shown in Tables 1 and 2. In the pT2 population, more than 97% of patients had clinical T2 or lower T-stage tumors. Nerve sparing was performed more aggressively in patients who were not evaluated at an MDTM than in those with an MDTM (33.2% vs. 10.0%, *p* = 0.0001). The PSM rate was significantly lower in cases evaluated at an MDTM than in those which were not (11.1% vs. 24.0%, *p* = 0.0067) (Table 1). In the pT3 population, 17% of cases evaluated at an MDTM and only 4% of those which were not had clinical T3 tumors (*p* = 0.0367) (Table 2). The PSM rates were approximately 50% in patients who were and were not evaluated at an MDTM, showing no significant difference (*p* = 0.9999), in contrast to the pT2 population.

**Table 1:** Patient clinical and pathological characteristics in pT2 cases

	Without MDTM	With MDTM	*p*-value
Number of patients	391	90	
Age, years (median)	69 (45–81)	69. (50–81)	0.4470
BMI, kg/m^2^ (median)	23.02 (16.6–31.8)	23.3 (16.7–35.2)	0.1779
PSA, ng/ ml (median)	7.2 (2.75–55.3)	7.2 (0.1–24.4)	0.2089
Prostate volume, ml (median)	28.7 (6.7–92.0)	31.8 (7.57–99.1)	0.0714
Clinical T stage, n (%)			
≤ T2	379 (96.9)	88 (97.8)	0.9999
T3	12 (3.1)	2 (2.2)	
Biopsy ISUP Grade Group, n (%)			
1	50 (12.8)	11 (12.2)	0.2244
2	110 (28.1)	28 (31.1)	
3	107 (27.4)	15 (16.7)	
4	100 (25.6)	27 (30.0)	
5	24 (6.1)	9 (10.0)	
Nerve-sparing			
None	261 (66.8)	81 (90.0)	0.0001
Unilateral	106 (27.1)	5 (5.6)	
Bilateral	24 (6.1)	4 (4.4)	
RARP ISUP Grade Group, n (%)			
1	16 (4.1)	2 (2.2)	0.1777
2	134 (34.3)	39 (43.3)	
3	165 (42.2)	31 (34.4)	
4	50 (12.8)	8 (8.9)	
5	26 (6.6)	10 (11.1)	
Surgical margin, n (%)			
Negative	297 (76.0)	80 (88.9)	0.0067
Positive	94 (24.0)	10 (11.1)	
Extraprostatic extension, n (%)			
Negative / x	388 (99.2)	90 (100.0)	0.9999
Positive	3 (0.8)	0 (0.0)	

BMI, body mass index; ISUP, International Society of Urological Pathology; RARP, robot-assisted radical prostatectomy

**Table 2:** Patient clinical and pathological characteristics in pT3 cases

	Without MDTM	With MDTM	*p*-value
Number of patients	75	29	
Age, years (median)	70 (47–76)	70 (55–79)	0.1768
BMI, kg/m^2^ (median)	23.9 (17.5–35.3)	23.5 (19.6–32.9)	0.9492
PSA, ng/ml (median)	9.0 (3.92–51.7)	7.0 (4.1–75.6)	0.2034
Prostate volume, ml (median)	27.0 (10.0–116.0)	32.9 (12.0–63.9)	0.7793
Clinical T stage, n (%)			
≤ T2	72 (96.0)	24 (82.8)	0.0367
T3	3 (4.0)	5 (17.2)	
Biopsy ISUP Grade Group, n (%)			
1	1 (13.3)	1 (3.5)	0.7430
2	13 (17.3)	4 (13.8)	
3	20 (26.7)	11 (37.9)	
4	24 (32.0)	8 (27.6)	
5	17 (22.6)	5 (17.2)	
Nerve-sparing			
None	66 (88.0)	28 (96.6)	0.1846
Unilateral	9 (12.0)	1 (3.4)	
Bilateral	0 (0.0)	0 (0.0)	
RARP ISUP Grade Group, n (%)			
1	0 (0.0)	0 (0.0)	0.5901
2	6 (8.0)	5 (17.2)	
3	33 (44.0)	11 (37.9)	
4	11 (14.7)	4 (13.8)	
5	25 (33.3)	9 (31.0)	
Surgical margin, n (%)			
Negative	37 (49.3)	14 (48.3)	0.9999
Positive	38 (50.7)	15 (51.7)	
Extraprostatic extension, n (%)			
Negative / x	70 (93.3)	28 (96.6)	0.9999
Positive	5 (6.7)	1 (3.4)	
Seminal vesicle invasion, n (%)			
Negative	50 (66.7)	20 (69.0)	0.9999
Positive	25 (33.3)	9 (31.0)	

### Predictors of PSM in pT2 population

Cox regression analyses were performed to further investigate the impact of MDTM on reducing the incidence of positive margins following RARP in pT2 patients (Table 3). Having PSA more than 7.0 ng/ml, Gleason’s grade 4 or 5 in the prostate biopsy, performing nerve sparing, and not conducting an MDTM were the risk factors for PSMs on univariate analysis. Multivariate analysis using these 4 factors revealed that a PSA level > 7 ng/mL (hazard ratio 2.2799) and conducting nerve sparing (hazard ratio 2.2619) were independent predictors of increased PSM risk, whereas conducting an MDTM (hazard ratio 0.4773) was an independent predictor of reduced PSM risk in the pT2 population.

**Table 3:** Univariate and multivariate analyses of different parameters for positive surgical margin in pT2 patient

		Univariate				Multivariate			
		Hazard ratio	95% CI		*p*-value	Hazard ratio	95% CI		*p*-value
			Lower limit	Upper limit			Lower limit	Upper limit	
Age (years old)	≥ 70	0.1689	0.9707	1.1772	0.1689				
	< 70	Ref							
PSA (ng/ml)	≥ 7.0	1.1300	1.0291	1.2408	0.0102	2.2799	1.4116	3.6823	0.0008
	< 7.0	Ref							
Prostate volume (ml)	≥ 30	0.9370	0.8526	1.0297	0.1774				
	< 30	Ref							
Biposy Gleason’s grade	4, 5	1.1063	1.0089	1.2131	0.0396	1.3289	0.7587	2.3276	0.3200
	1–3	Ref							
Body mass index	≥ 23	0.9934	0.9043	1.0913	0.8904				
	< 23	Ref							
Nerve sparing	Yes	1,8766	1.3436	2.6209	0.0004	2.2619	1.3318	3.8416	0.0025
	No	Ref							
Conducting MDTM	Yes	0.4622	0.2510	0.8509	0.0043	0.4773	0.2331	0.9773	0.0431
	No	Ref							

CI, confidence interval; MDTM, multidisciplinary team meeting

### Characteristics of PSM

**Fig. 1:**
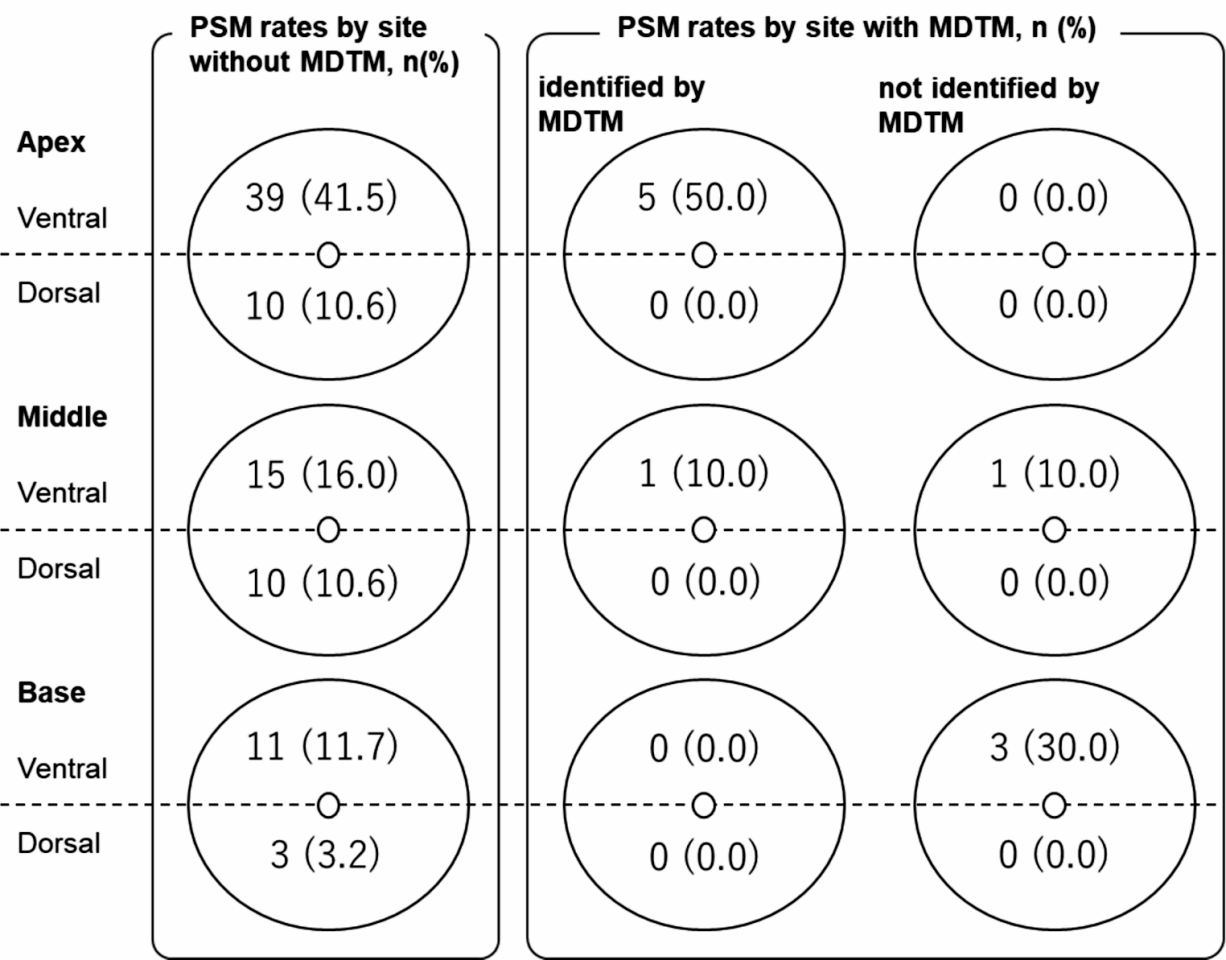
PSM rates by site in pT2 patients with and without MTDM. PSM rates by site were described for the three groups: sites identified without MDTM, sites identified by MDTM, and sites that could not be identified by MDTM. Six of the 94 PSM cases without MDTM (6.4%) had unknown sites. MDTM, multidisciplinary team meeting; PSM, positive surgical margin

Next, we investigated whether there were differences in the PSM sites between pT2 patients with and without MTDM (Fig. 1). The site with the highest PSM rate, with or without MDTM, was the ventral side of the apex. Of the 10 PSM cases with MDTM, all were detected from the ventral prostate, and six were detected at locations noted in the MDTM. Of these, five were detected on the ventral side of the apex (middle part of Fig. 1). However, 1 case and 3 cases in which PSMs were detected in the middle and base of the prostate, respectively, were not be noted at the MDTM (right part of Fig. 1). A PSM was not detected on the dorsal side in patients who were evaluated at an MDTM, whereas it was detected in 24.4% (23 of 94 PSM cases) of the patients who were not evaluated at an MDTM.

Finally, we assessed whether surgeon experience affected the effectiveness of the MDTM in terms of the incidence of PSM (Table 4). In the pT2 and pT3 populations, 255 and 17 patients, respectively, underwent RARP by experienced surgeons, whereas 226 and 12 patients, respectively, underwent RARP by non-experienced surgeons. The PSM rates in cases evaluated at an MDTM did not show statistically significant differences between non-experienced and experienced surgeons in the pT2 and pT3 populations (*p* = 0.9999 and 0.4621, respectively).

**Table 4:** Differences in PSM rates of cases with MDTM between experienced and non-experienced surgeons

		Non-experienced	Experienced	*p*-value
pT2	no. of PSM / total (%)	5 / 226 (11.9)	5 / 255 (10.4)	0.9999
pT3	no. of PSM / total (%)	5 / 12 (41.7)	10 / 17 (58.8)	0.4621

MDTM, multidisciplinary team meeting; PSM, positive surgical margin

## Discussion

MDTMs have increasingly been implemented in cancer care, including PCs worldwide, to ensure timely, accurate, and evidence-based diagnosis and treatment plans [15]. The effectiveness of MDTM in reducing the PSM rate has been reported in other cancers. The MDTM on MRI and implementation of a preoperative treatment strategy result in significantly reduced positive histopathological audits of positive circumferential resection margins in patients with rectal cancer [16]. The favorable effects of MDTMs on survival, recurrence or metastasis, mortality, and other patient-related outcomes have been reported in colorectal, breast, and lung cancers; however, it is unclear whether this also results in improved patient outcomes in PCa [15, 17].

The present study identified the advantages and disadvantages of the preoperative MDTM for patients with PCa. The PSM rate in the pT2 population significantly improved in patients who received preoperative MDTM (Table 1), and MDTM reduced the risk of PSM occurrence by 54% according to our multivariate analysis (Table 3). The PSM rates after MDTM were equally low between experienced and non-experienced surgeons in the pT2 and pT3 populations. A previous study investigating the risk factors for PSM after RARP revealed that the surgeon’s experience had no effect on PSM rates in pT3b tumors. However, it was significantly associated with lower PSM rates in pT2 and pT3a tumors [1]. Functional and oncological outcomes after RP have been shown to depend mostly on surgeons’ experience and hospital volume [18]. Nevertheless, the majority of patients require and research the new, that is, the “robotic approach” [19]. Although all PCa patients underwent RARP in this study, the number of RARP cases is not very large in our institution, and the experience of each surgeon is dispersed. Thus, the preoperative MDTM can be an important factor that complements the surgeon’s experience.

In the pT2 population, the reason for the lower frequency of nerve sparing in patients evaluated at an MDTM might be that MDTM prompted surgeons to consider more strictly which cases qualify for nerve sparing. Methods to avoid the risk of increased PSM due to nerve sparing (i.e. NEUROSAFE) (20) should be considered.

PSM on the dorsal side was not detected in patients evaluated at an MDTM, whereas it was detected in 24% of the patients who were not evaluated at an MDTM, suggesting that MDTM might be useful for PCs in dorsal lesions. Several studies report that PSMs are most common at the apex [6]. We could not completely avoid PSMs, especially at the apex, even when the locations were noted at the preoperative MDTM. Furthermore, in the ventral-middle and basal locations, PSMs that could not be identified even with the MDTM were detected, suggesting the limitations of the MDTM.

PSM rates in pT3 cases were not different from those in previous reports [10] with or without MDTM, while only 17.2% of pT3 cases were diagnosed with clinical T3 even after conducting an MDTM. Recent evidence suggests that MRI could improve the accuracy of the diagnostic assessment of PCa [21], and is regarded as the best available imaging tool for assessing the T stage in clinical practice [22]. Although the T3 stage is a significant pathological characteristic that increases the likelihood of PSMs and biochemical recurrence [23], identifying the T3 stage using MRI is often considered subjective [22]. A more accurate method for diagnosing clinical T3 disease is required.

The present study had some limitations. Few patients in this study were evaluated at an MDTM. The number of PSM among patients evaluated at an MDTM was small, which may have affected the statistical analyses. The pT2 patients with MDTM had significantly lower rates of nerve preservation. Thus, the differences in urinary incontinence or erectile dysfunction between those with or without MDTM should be investigated near future.

In conclusion, the present study revealed for the first time that preoperative MDTM improves the incidence of positive margins in pT2 PCa. Considering that 97.8% of pT2 patients had a preoperative diagnosis of clinical T2 stage and 82.8% of pT3 cases were clinical T2, MDTM should be conducted for all clinical T2 patients with localized PCs, although the significance of MDTM is less for pT3 cases. Considering that PSM is an independent predictor of PCa-specific mortality [3, 4], our outcomes may have long-term implications for patient survival expectancy after RARP. Therefore, further long-term observations are required.

## Data Availability

Data are available from the corresponding author upon reasonable request.
